# The Molecular Mechanisms Underlying Onset and Progression of Liver Cancers

**DOI:** 10.3390/cancers15174383

**Published:** 2023-09-01

**Authors:** Clara Le Breton, Cédric Coulouarn

**Affiliations:** Inserm, University of Rennes, UMR_S 1242, OSS (Oncogenesis Stress Signaling), Centre de Lutte Contre le Cancer Eugène Marquis, 35042 Rennes, France; clara.lebreton@univ-rennes1.fr

Liver cancers, including hepatocellular carcinoma (HCC) and cholangiocarcinoma (CCA), are deadly cancers that have risen in frequency globally and have limited curative therapeutic options. Liver carcinogenesis is a multistep process associated with the accumulation of molecular abnormalities. These abnormalities, including DNA mutations, gain/loss of heterozygosity, and/or epigenetic alterations, contribute to the modulation of cellular signaling pathway activities involved in tumor onset and progression. A better understanding of molecular mechanisms underlying liver carcinogenesis should help in designing innovative therapeutic strategies and identifying innovative biomarkers for the management of patients suffering from liver cancer. The goal of this Special Issue of *Cancers* is to provide an overview of these molecular mechanisms. It is based on review and research articles that report emerging experimental evidence of the multifaceted regulation of signaling pathways involved in liver cancer from an epigenetic, transcriptional, and post-translational perspective.

Epigenetics encompasses heritable changes in gene expression that occur without altering the DNA sequence itself, including DNA methylation and histone modifications. In this context, Gulati and colleagues reported that the chromatin status of so-called cancer-enhancing genomic regions (CEGRs) and aggressive liver cancer domains (ALCDs) can regulate the transcription of key oncogenes in pediatric liver cancer hepatoblastoma [1]. Most notably in their research, they demonstrated that the phosphorylation of β-catenin at Ser675 (ph-S675-β-catenin) induces the formation of a complex with TCF4 transcription factor leading to the activation of CEGRs/ALCDs through a p300-dependent acetylation of histones. This study demonstrates the essential role of the ph-S675-catenin-TCF4-CEGRs/ALCDs axis in the development of aggressive hepatoblastoma [1]. While reviewing emerging factors and treatment options in nonalcoholic fatty liver disease (NAFLD)-related HCC, Zhang and Yang highlight the role of DNA methylation in modulating the expression of specific genes at different stages of NAFLD-related HCC onset and progression [2]. The authors also highlight non-coding RNAs, including microRNAs (miRNAs) and long non-coding RNAs (lncRNAs), as epigenetic factors associated with the development of NAFLD-related HCC [2]. In particular, the over-expression of miR-21, a well-known pro-oncogenic miRNA in several cancers, is highlighted for its ability to contribute to the broad spectrum of NAFLD features, such as liver steatosis, inflammation, and fibrosis. The authors also report the involvement of lncRNAs in carcinogenesis, specifically related to endoplasmic reticulum and oxidative stress but also to pro-inflammatory signaling pathways [2]. Similarly, Recalde and colleagues connect epigenetic alterations and the progression of liver cancer by reporting the promoter hyper-methylation of 35 lncRNAs that compromise cell differentiation in HCC [3]. Down-regulation of these lncRNAs correlates with more advanced histological grades and a decreased overall survival of patients [3].

From a transcriptional point of view, gene expression is closely regulated by the splicing step to produce mature transcripts. Alternative splicing during pre-mRNA maturation results in changes that might affect proteins involved in different aspects of cancer biology. In their review article, Marin and colleagues report abnormalities in the splicing machinery or genetic mutations inducing an alternative splicing that occurs during liver cancer progression, which lead to truncated or aberrant proteins [4]. They also describe the altered expression of spliceosome components and associated regulating proteins on the outcome of patients with liver cancer [4]. Those proteins are involved in several hallmarks of cancer, resulting, notably, in abnormal cell proliferation, cytoskeleton disorganization, migration, invasion, and multidrug resistance [4]. Apart from alternative splicing, several papers also shed light on different gene expression signatures derived from liver cancer cells. On this point, Desoteux and colleagues identify genes differently expressed according to the differentiation status of tumor hepatocytes, including a minimal subset of seven genes predicting a poor prognosis in patients with HCC [5]. O’Brien and colleagues investigate the importance of estrogen receptor 1 (Esr1) in the development of HCC in female mice [6]. Furthermore, they show that Esr1 is a crucial mediator of tumor outcome, which protects female mice against liver tumor development. They also report a signature of six genes highly dysregulated by Esr1 deficiency and implicated in HCC. This work indicates that Esr1 mediates liver cancer risk, and its control of sex-specific liver gene expression involves cells other than hepatocytes [6]. 

Deleted in liver cancer 1 (*DLC1*) is a well-known tumor suppressor gene whose allele is lost in 50% of liver cancers. Restoration of DLC1 expression induces cellular senescence, but this principle is hardly amenable to direct clinical therapeutic targeting. Thus, Schreyer and colleagues search for a druggable target gene that mediates the effects of DLC1 on senescence induction [7]. Tetraspanin 5 (TSPAN5), which plays a crucial role in promoting the growth, migration, and invasion of HCC cells, is identified as a novel gatekeeper of DLC1 and a novel therapeutic target for the treatment of HCC characterized by *DLC1* loss [7]. Aside from this cellular network, numerous signaling cascades promote HCC onset and progression. Hedrich and colleagues focus on Tyro3, Axl, and MerTK receptor tyrosine kinases, which constitute the TAM family [8]. They review the role of these receptors, emphasizing their implications in immunity, fibrogenesis, and chemoresistance and their potential as therapeutic targets in HCC [8]. 

Post-translational modifications are also critical functional features involved in human carcinogenesis. For instance, Liu and colleagues report the role of O-GlcNAcylation in promoting a malignant HCC phenotype [9]. O-GlcNAcylation is controlled by writers and erasers, including O-GlcNAc transferase (OGT) and O-GlcNAcase (OGA), respectively. An increase in total O-GlcNAc correlates with advanced HCC phenotypes. The authors demonstrate that RANBP2, one of the SUMO E3 ligases, SUMOylates CEBPa transcription factor and down-regulates OGA transcription while not affecting OGT, thus reducing the levels of erasers [9]. In this context, Yuan and colleagues review the role of protein SUMOylation in HCC [10]. SUMOylation impacts many aspects of tumor cell biology, including DNA repair, transcription, protein stability, and cell cycle progression. Accordingly, this post-translational modification affects HCC development by enhancing the growth and viability of tumor cells. SUMOylation also contributes to metastasis and drug resistance by modulating tumor cell migration and invasion and by remodeling the tumor microenvironment [10].

Altogether, the research and review articles included in this Special Issue illustrate the diversity of molecular mechanisms involved in liver tumor onset and progression, from epigenetic to post-translational modifications. These articles highlight molecular targets that should be further investigated to build up new therapeutic orientations and to identify clinically relevant biomarkers. Advances in this field may be applied for better management of patients with HCC in terms of diagnosis, patient care, and chemoresistance fighting.
